# Atlas of Venereal and Skin Diseases

**Published:** 1888-05

**Authors:** 


**Atlas of Venereal and Skin Diseases,** containing original illustrations and selections from the plates of Kaposi and Neuman, of Vienna; Hutchinson, of London; Ricord and Vidal, of Paris, and others, with original text by PRINCE A. MORROW, M. D., Clinical Professor Venereal Diseases and Lecturer on Dermatology, University of the City of New York. Wm. Wood & Co., New York. Mattison, East Eagle street, Buffalo.

In our last number, we acknowledged the receipt of Parts I. and II. of this magnificent work. Fasiculus III. is just received. It contains the following plates: Chancre of fore finger, with syphilide of palm; chancre of nipple, erythematous syphilide, milliary syphilide, papular syphilide, papulo pustular syphilide. The plates are beautiful, and we can only repeat the expressions of admiration which every issue of this great work has elicited from all who have seen them.

